# Evaluation of the Clinical Utility of Genomic Profiling to Inform Selection of Clinical Trial Therapy in Salivary Gland Cancer

**DOI:** 10.3390/cancers14051133

**Published:** 2022-02-23

**Authors:** Samuel Rack, Laura Feeney, Brindley Hapuarachi, Helen Adderley, Laura Woodhouse, Guy Betts, George J. Burghel, Kevin J. Harrington, Robert Metcalf

**Affiliations:** 1 Department of Medical Oncology, The Christie Hospital NHS Foundation Trust, Manchester M20 4BX, UK; sam.rack@nhs.net (S.R.); h.adderley@nhs.net (H.A.); laura.woodhouse3@nhs.net (L.W.); 2 The Northern Ireland Cancer Centre, Belfast City Hospital, Lisburn Road, Belfast BT9 7AB, UK; laura.feeney@nhs.net; 3 Sheffield Teaching Hospitals NHS Foundation Trust, Glossop Road, Broomhall, Sheffield S10 2JF, UK; sonal.hapuarachi@nhs.net; 4 Department of Adult Histopathology, Manchester University NHS Foundation Trust, Oxford Road, Manchester M13 9WL, UK; guy.betts@mft.nhs.uk; 5 North West Genomic Laboratory Hub, Manchester Centre for Genomic Medicine, Manchester University NHS Foundation Trust, Oxford Road Rd, Manchester M13 9WL, UK; george.burghel@mft.nhs.uk; 6 The Royal Marsden NHS Foundation Trust, Fulham Rd., London SW3 6JJ, UK; kevin.harrington@icr.ac.uk

**Keywords:** salivary gland cancer, adenoid cystic carcinoma, salivary duct carcinoma, molecular profiling, biomarker, clinical trials

## Abstract

**Simple Summary:**

Salivary gland cancer is rare and there is a need to develop new and effective drug therapies. New drugs are in development in the recurrent and metastatic setting that target specific changes within a cancer, some of which can be detected through sequencing of the cancer DNA. This study addressed how useful the DNA sequencing of cancer samples is to inform the decision of which drug therapy within a trial is the best match for each individual patient. We found that using focused DNA panels, including small numbers of genes, helped to match just over one in four patients with salivary gland cancer to drug therapies. The matching rate of the focused panel varied by subtype and was least useful in adenoid cystic carcinoma (ACC), at 7%. However, in ACC, larger gene panels had added value, identifying matched trial therapies in 40% of cases.

**Abstract:**

For most patients with salivary gland cancer, there are no effective standard systemic therapies. Although clinical trials of biomarker-led drug therapies have delivered significant recent advances, there remains a need to understand the clinical utility of genomic profiling of cancer as a means to match patients with recurrent or metastatic salivary gland cancer to clinical trial therapies. In total, 209 patients with salivary gland cancers were profiled with 24 gene (*n* = 209)) and >325 gene (*n* = 32) DNA-based next-generation sequencing panels. A retrospective systematic evaluation was performed to identify the frequency of available matched drug therapies within clinical trials based on the results. The matches were then stratified based upon the level of evidence supporting the drug–biomarker combination being investigated using the ESMO Scale for Clinical Actionability of Molecular Targets (ESCAT) to determine the strength of the clinical rationale for each gene–drug match identified. DNA-based next generation sequencing (NGS) analysis was successful in 175/209 (84%) patients with salivary gland cancer. Using the 24-gene NGS panel, actionable alterations were identified in 27% (48/175) patients. Alterations were most frequent in salivary duct carcinoma (88%) characterized by TP53 and/or PIK3CA mutations, with matched trials available for 63% (10/16). In ACC, biomarker-matched trials were available for 7% (8/115), and no genomic alterations were found in 96/115 (83%) of ACC patients. TP53 was the most frequently altered gene across all subtypes; however, there were no trials recruiting based on TP53 status. In 32 ACC patients with no genomic alterations using the 24-gene panel, a broader (>325 gene) panel identified alterations in 87% (27/32) of cases with biomarker-matched trials available in 40% (13/32) cases. This study identified that genomic profiling using focused (24-gene) NGS panels has potential utility in matching to trial therapies for most patients with non-ACC salivary gland cancer. For patients with ACC, broader genomic profiling has demonstrated added clinical utility. We describe the application of an approach to classification of levels of evidence which may be helpful to inform the clinician and patient decision making around the selection of clinical trial therapies.

## 1. Introduction

Salivary gland cancer (SGC) is a rare disease comprising over 24 histopathological subtypes [1]. Although many patients are cured following surgical resection with or without adjuvant radiation, consensus guidance on the optimum management of recurrent or metastatic disease is based on limited evidence and hindered by the relative paucity of clinical trials incorporating these patients [2].

Although some patients may derive meaningful benefit from cytotoxic chemotherapy [3], there remains limited trial evidence to support its routine use. The most significant recent advances have come from clinical trials of biomarker-directed drug therapies [4,5,6,7,8,9,10,11,12]. For example, the amplification of Erb-B2 receptor tyrosine kinase 2 (ERBB2/HER2) protein overexpression is seen in 20–30% of cases of salivary duct carcinoma [13] and there is trial data to support the efficacy of HER2-targeting therapy with either trastuzumab and docetaxel [11], trastuzumab and pertuzumab [12] or trastuzumab-emtansine [10] in recurrent or metastatic ERBB2-amplified/HER2-overexpressing disease. In addition to HER2, the androgen receptor (AR) is overexpressed in most patients with salivary duct carcinoma (SDC) [13]. There are clinical trial data showing the efficacy of either androgen deprivation therapy with bicalutamide [13] and enzalutamide [9] and combined androgen blockade with bicalutamide and triptorelin [8] in recurrent or metastatic AR-overexpressing salivary duct carcinoma.

The other subtype of salivary gland cancer which has been most impacted by the development of biomarker-led therapy is secretory carcinoma. Chromosomal rearrangements resulting in fusions of the Neurotrophic Tyrosine Receptor Kinase (NTRK) gene are described in around 90% of cases of secretory carcinoma [14] and there are data showing the efficacy of entrectinib [4] and larotrectinib [5] in NTRK-rearranged tumours from any primary site, including NTRK-rearranged salivary gland cancers. Previous studies in SGC identified TP53 and the PI3K pathway as the most commonly altered through point mutations across SGC subtypes. Mutations in ERBB2, EGFR, and BRAF have previously been detected at low frequencies and have the potential to match patients to targeted therapies [15].

Aside from these few examples of biomarker-directed therapies relevant to salivary gland cancers, in order for clinicians to engage in informed discussion with their patients on the potential role for genomic profiling, there is a need to better understand the clinical utility of these approaches. In addition, clinicians increasingly encounter situations in which patients attend consultations seeking an interpretation of genetic sequencing data on their own tumours that they have obtained from commercial vendors. We, therefore, sought to evaluate the frequency with which DNA-based next-generation sequencing (NGS) yields additional information to aid selection of clinical trial drug therapies for a cohort of patients with salivary gland cancer and to judge the strength of the underlying rationale for individual biomarker–drug matches in this population.

## 2. Materials and Methods

### 2.1. Patient Consent and Clinical Data Collection

We performed a retrospective cohort study on 209 patients with salivary gland cancers who underwent clinical review at a tertiary cancer centre (The Christie NHS Foundation Trust, Manchester, UK) from April 2017 to December 2020. Patients provided informed consent for the collection of genomic, clinical and demographic characteristics. This study was granted research ethics approval under the Manchester Cancer Research Centre Biobank Research Tissue Bank Ethics (NHS NW Research Ethics Committee 18/NW/0092) and was performed in accordance with the Declaration of Helsinki.

### 2.2. Next-Generation Sequencing

For all patients, a focused DNA-based NGS panel was performed including 24 cancer-related genes through the course of this study. The genes included and regions of each gene covered are shown in Supplementary Table S1. DNA was extracted from archival FFPE samples, and samples were requested to have a minimum tumour content of 20% and analysed using a Qiagen GeneRead DNAseq Targeted Panel V2 in the National Health Service Northwest Genomics Laboratory Hub. A median exon coverage depth of >350× was performed and a customised bioinformatic pipeline was validated to detect single nucleotide variants and indels (<40 bp) to 4% mutant allele frequency. Where identified, variants were classified using Cancer Variant Interpretation Guidelines UK [16] and tiering from the joint Consensus Recommendation of the Association for Molecular Pathology, American Society of Clinical Oncology, and College of American Pathologists [17] with reference to publicly available resources including Catalogue Of Somatic Mutations In Cancer v19, and other subscription-based resources including Human Gene Mutation Database Professional (Qiagen). In a secondary analysis, on samples from 32 patients with adenoid cystic carcinoma, in whom no alterations were identified using the focused panel, repeat analysis was performed using a broader DNA-based NGS panel. For these samples, the commercial Foundation Medicine assay was performed in a Clinical Laboratory Improvement Amendments (CLIA)-certified laboratory and accredited by the College of American Pathologists. Tumour DNA underwent hybrid capture for the entire coding region and select introns from genes frequently mutated or rearranged in cancer (including >325 selected cancer-related genes through the course of this study). Illumina HiSeq NGS was performed with a median exon coverage depth of >500× to identify single-nucleotide variants, indels, copy number alterations and select gene rearrangements (as described in [18]). Summary findings of NGS data were visualised on cBioPortal [19,20].

### 2.3. Systematic Evaluation of Genomic Biomarker and Drug Trial Matches

To link genetic variants to drug therapies within clinical trials, the 24 genes included in the focused panel were queried in the public ally available databases OncoKB [21] and MyCancerGenome.org to identify pre-existing biomarker–therapy matches and biomarker-led studies, respectively. To identify preclinical evidence for biomarkers associated with response to targeted therapies, literature searches of PubMed were undertaken using the search terms “GENE NAME” and (“predictive biomarker” OR “Targeted therapy” OR “Response” OR “sensiti*”). To determine whether any clinical trials were available in the UK incorporating these therapies and open to patients with salivary gland cancers, a search was performed on the clinicaltrials.gov database for trials open to recruitment to patients with salivary gland cancers or any solid tumour type using therapies established from the above process with sites active in the UK from 2016 to December 2020.

### 2.4. Classification of Level of Evidence

Genomic findings were ranked using the European Society of Clinical Oncology (ESMO) Scale for Clinical Actionability of Molecular Targets (ESCAT) scoring system [22] independently by two co-authors (S.R. and L.W.). When different scores were allocated, a further review was undertaken by R.M. and a final consensus reached. The ESCAT scaling defines six tiers of clinical evidence supporting the actionability of genomic–drug matches as follows: Level I, genomic–drug matches ready for implementation in routine clinical decisions; level II, genomic–drug matches as investigational targets that are likely to define a patient population that benefits from a targeted drug but additional data are needed; level III, genomic–drug matches with clinical benefit previously demonstrated in other tumour types or for similar molecular targets; level IV, genomic–drug matches with preclinical evidence of actionability; level V, genomic–drug matches with evidence supporting co-targeting approaches; and level X, genomic findings with lack of evidence for actionability. For the ESCAT classification of each gene under investigation, the highest score was taken when different specific variants within the same gene carried different scores.

## 3. Results

### 3.1. Patient Characteristics

A total of 209 patients with salivary gland cancer reviewed at a single tertiary cancer centre between 2017 and 2019 were included in this study. The clinical characteristics of the patients are shown in Table 1. Consistent with a population of patients seeking clinical trial therapies, the median age was 51 years (range: 23–78 years) and almost all patients (91%) had radiological confirmation of recurrent or metastatic disease with a measurable component at the time of review. In total, 44% of patients had tumours arising in the minor salivary gland consistent with the relatively high frequency of disease recurrence in this population [23]. ACC was the most frequent histopathology (68%), as would be expected given the relatively high risk of recurrence in this subtype. Of the remaining patients with non-ACC SGC, adenocarcinoma not otherwise specified (NOS) and SDC were most frequent.

### 3.2. Next-Generation Sequencing with 24-Gene Targeted Panel

#### 3.2.1. Systematic Evaluation of Matched Drug Therapies within Clinical Trials

To facilitate personalised clinical trial selection, a focused DNA-based NGS panel including 24 genes frequently associated with somatic mutations in cancer (Table S1) was applied to FFPE tumour samples from 209 patients with salivary gland cancers being reviewed to consider clinical trial therapies. To determine the utility of this panel to guide clinical trial selection, we performed a systematic evaluation of genomic biomarker–drug trial matches. Table 2 summarises the genes included in this panel and the matched drug therapies available through the course of this study.

Although all of the genes included in this panel had the potential to match patients to drug therapies, through the duration of this study, matched drug therapies within trials were open to SGC/solid malignancy patients for 63% (15/24) of the sequenced genes. To classify the level of evidence supporting the genomic biomarker under investigation, genomic findings were ranked using the ESCAT scoring system [22]. None of the genomic alterations was classed as level 1, defined as the drug–target match being associated with improved outcome in clinical trials in the specific tumour type. The highest ESCAT score was for PIK3CA at level 2b, as clinical trial results of drugs targeting this pathway have demonstrated a signal of increased radiological response rate without data showing a meaningful overall survival benefit [38].The remainder were classed as level 3 (12/15), defined as demonstrating clinical benefit in other tumour types or in similar variants, or level 4a (2/15), defined as actionability of target predicted in preclinical in vitro or in vivo models.

**Figure 1:**
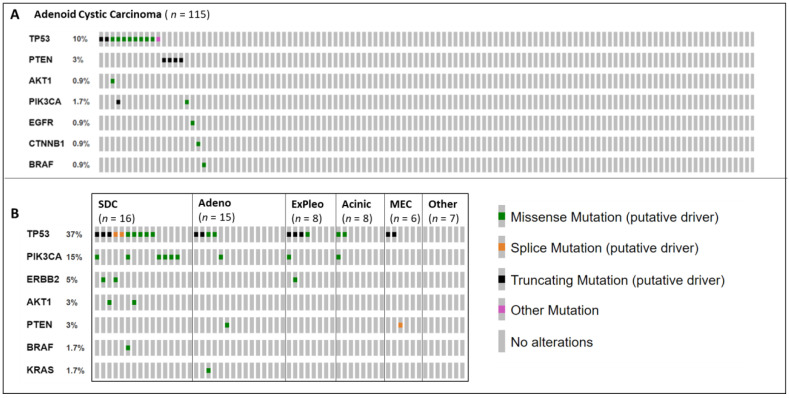
Results of DNA-based next-generation sequencing from patients with salivary gland cancer (*n* = 175) using 24-gene NGS panel. (**A**) Adenoid Cystic Carcinoma (ACC). (**B**) Patients with other (non-ACC) histopathological subtypes. Other includes neuroendocrine carcinoma (*n* = 1), secretory carcinoma (*n* = 1) and NUT carcinoma (*n* = 1). Individual patient results are represented by a column of vertical bars. Only genes in which alterations were detected are shown. Detection of genomic alterations are indicated by coloured bars, and the absence of alterations is indicated by grey bars.

Applying the 24-gene NGS panel to a consecutive series of 209 patients with salivary gland cancer considering trial therapies, the analysis through NGS was successful in 175 cases (84%). The remainder failed due to insufficient or poorly preserved DNA. Figure 1 shows the results of the focused NGS analysis on 175 patients with salivary gland cancer. Sixty-three variants of significance were identified in 48 patients (27%). The most frequently altered genes were PIK3CA and TP53, which made up 61% (39/63) of all alterations identified. The frequency of alterations was lowest for patients with ACC (19/115, 17%) compared with non-ACC SGC (29/60, 50%). The highest frequency of alterations seen using this approach was for patients with SDC which was characterised by the presence of TP53 and/or PIK3CA mutations in 14/16 cases. Patients with myoepithelial, neuroendocrine (*n* = 1), NUT (*n* = 1) and secretory (*n* = 1) carcinomas had no identifiable mutations with this panel.

#### 3.2.2. TP53

TP53 alterations were the most frequent finding, with 34 mutations identified in 33 samples (Table 3). These were missense mutations in 19/34, and 12/34 were truncation mutations. One sample had a silent mutation, TP53 c.375G > A p.(Thr125Thr). However, this variant has been shown to lead to in intron inclusion between exons 4 and 5 and is therefore pathogenic. Depicted as “other” in Figure 1, further mutations occurred at splice sites, resulting in pathogenic changes. Although TP53 alterations were the most frequent finding, the drug–biomarker combinations are under investigation in trials based upon pre-clinical (level 4a evidence), and there were no open trials with a rationale for the inclusion of patients with TP53 mutations through the course of this study. Level 4a was allocated based on preclinical data with ATR inhibitors. TP53-mutant tumours were shown to have increased sensitivity to ATR inhibitors in combination with chemotherapy or radiotherapy [42].

#### 3.2.3. PIK3CA/AKT/PTEN

In total, 50% (8/16) of patients with SDC had mutations in PIK3CA/AKT/PTEN, conferring the hyperactivation of the PI3K signalling pathway. In ACC, the frequency was 5% (6/115). In PIK3CA, 12 gain-of-function variants were detected in 11 patients (Table 3). As such, 2/115 ACC patients (2%) could have been matched to trials based on their PIK3CA mutation status with level 2b evidence. Level 2b was attributed to PIK3CA mutations based on preliminary data published from basket trials including PI3K inhibitors, showing a response rate of 16% and stable disease rate of 66% at 6 months in patients with tumours harbouring PIK3CA mutations [38]. The utility of PIK3CA rises to 14% (9/63) when applied to nonadenoid cystic salivary gland cancer and to 37% (6/16) for patients with salivary duct carcinoma. However, in non-ACC SGC, the evidence level is 3a based on the efficacy of PIK3CA inhibitors in breast cancer [43]. One case of apelisib plus androgen deprivation in SDC is reported, resulting in significant benefits [44].

AKT1 gain-of-function (E17K) variants were identified in 1% (1/115) of ACC and 13% (2/16) of SDC patients. AKT1 inhibitors have shown higher response rates in a basket trial in patients with AKT1 E17K-mutated tumours; however, no SGC patients were included in this trial and they are, therefore, ranked as 3a.

Loss-of-function PTEN variants were observed in 3% (4/115) of ACC patients and 3% (2/63) of non-ACC SGC patients. Clinical responses have been seen in the early phase trials of PI3Kb inhibitors in tumours with PTEN loss in prostate cancer [39] and gastric cancer [45], giving PTEN a 3a ranking.

#### 3.2.4. Receptor Tyrosine Kinases—ERBB2 and EGFR

Pathogenic ERBB2 mutations were identified in three patients with salivary gland cancer (Table 3), which provides a match to trial therapies with level 3a/3b evidence. Level 3b is applied to SDC as there is evidence of efficacy of anti-HER2 therapy in HER2-amplified SDCs [13] and in other SGC subtypes. It was classified as 3a as a result of the efficacy of anti-HER2 therapies in lung cancer patients with gain-of-function mutations [46]. Again, this alteration was most frequent in patients with salivary duct carcinoma (2/16, 13%).

EGFR was mutated in one patient with adenoid cystic carcinoma (Table 3). The variant was c.2369C > T p.(Thr790Met), which is a commonly acquired variant that confers resistance to most EGFR tyrosine kinase inhibitors, but is sensitive to Osimertinib [30]. This matched these patients to trial therapies with level 3a evidence due to the efficacy data for non-small-cell lung cancer.

#### 3.2.5. Others—BRAF, KRAS and CTNNB1

Two BRAF alterations were identified in patients with adenoid cystic carcinoma and salivary duct carcinoma (Table S2). Both were class 3 gain-of-function mutations. Class 3 BRAF mutations are characterized by a dependence on RAS signalling and are hypothesized to be sensitive to RAS inhibition. While BRAF V600E would be classified as 3a, BRAF class 3 mutations are classed as X as they have no kinase activity themselves and are dependant on upstream oncogenic signalling and commonly co-occur with NF1 deletions [47]. A single KRAS mutation was identified in a patient with an adenocarcinoma; this missense mutation resulted in KRAS gain of function. The variant identified was not G12C and, as such, there is only preclinical evidence of increased sensitivity to MEK/ERK inhibitors. Thus, this variant was classified as 4a [36].

CTNNB1 codes for beta-catenin: One variant in an ACC patient was detected. Preclinical data have shown CTNNB1 variants to confer increased sensitivity to CBP/Betacatenin inhibitors, and so this variant was therefore classed as 4a [28].

### 3.3. Biomarker-Matched Trial Availability

Through the course of this study, using the 24-gene panel, 7% (8/115) of ACC patients could be matched to a biomarker-led clinical trial based on the molecular screening results. In 6% (7/115) of cases, this was due to variants conferring the hyperactivation of the PI3K pathway (AKT1, PIK3CA and PTEN variants), who could be treated with AKT1 inhibitors, such as Capivasertib (NCT0122631, NCT02338622). The remaining ACC match was for a patient with an EGFR mutation, who could have been matched to AFM24 (NCT04259450).

In SDC, 63% (10/16) could be matched to biomarker-led trials. A total of 50% (8/16) were, again, matched to AKT1 inhibitors such as Capivasertib and 13% (2/16) to HER-2 inhibitors such as Neratinib (NCT01953926) or TAS0728 (NCT03410927).

Matched trial therapies were available for 20% (3/15) of patients with adenocarcinoma (NOS), 13% (2/15) to AKT1 inhibitors (NCT01226316, NCT02338622) and 7% (1/15) to RAF/MEK inhibitors, based on a KRAS mutation through NCT02407509. The relatively small number of patients included with other non-ACC subtypes limits the analysis. However, matched trial therapies were available for 25% (2/8) of patients with carcinoma ex-pleomorphic adenoma, 20% (1/8) with acinic cell carcinoma, and 16% (1/6) with muco-epidermoid carcinoma.

### 3.4. Next-Generation Sequencing with 350+ Gene Targeted Panel

**Figure 2:**
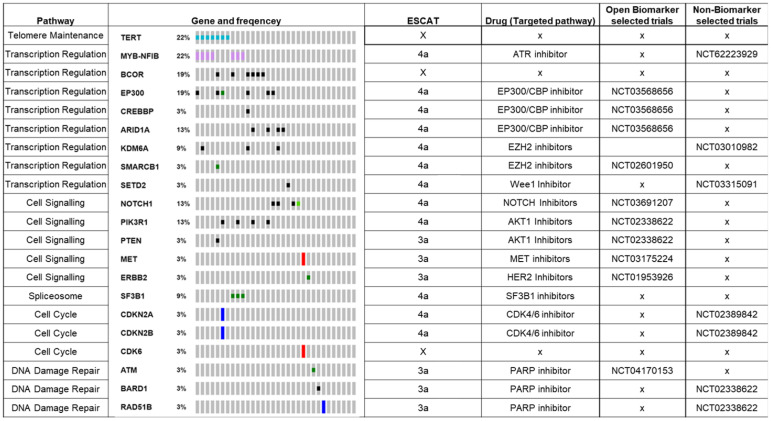
Results of 325-gene NGS panel in patients where no mutation was found in the focused NGS panel. Studies providing the rationale behind ESCAT classification can be found in Supplementary Table S2 [37,39,48,49,50,51,52,53,54,55,56,57,58,59]. Selected mutations are included in the figure, including all mutations with biomarker-stratified trials; the full list is in Supplementary Table S3.

As 81% of ACC patients (94/115) had no genetic alterations identified with the 24-gene panel, we next sought to determine the additional utility of applying a broader NGS panel in this cohort. We, therefore, re-analysed the FFPE tumour samples from 32 ACC patients in whom no variants had been detected on the focused NGS panels using a commercially available (>325-gene) NGS panel. Additional genomic findings were detected in 27/32 patients (84%). Figure 2 summarises the genetic alterations identified through this approach, and the full list of variations can be found in Supplementary Table S2. ESCAT scores were allocated to genes where a variant was detected; references can be found on Supplementary Table S2.

Additional utility was gained in 40% (13/32) of patients with regard to being able to match them to a biomarker-stratified clinical trial.

MYB-NFIB fusions and TERT promoter mutations were the most common alterations, with 22% (7/32) and 22% (7/32), respectively. There are no current compounds being developed to target TERT promoter mutations. MYB overexpression as a result of fusion transcripts is the hallmark of ACC, ATR has been shown to be downstream of MYB and in preclinical studies of ACC models, treatment with ATR inhibitors has led to apoptosis and growth inhibition [48].

Genes coding for chromatin modifiers (EP300, ARID1A, KDM6A, BCOR, CREBBP, SETD2, SMARCB1) were altered in 44% (14/32) of patients, with 13% (4/32) having more than one gene altered. EP300/CREBBP inhibitors are being trialled in EP300-, ARID1A- and CREBBP-deficient tumours [49].

NOTCH1 was altered in 13% (4/32), and in three cases these were activating mutations. These can be targeted by gamma secretase inhibitors, which are in phase 2 trials in ACC. AL101 achieved a response rate of 9% with a disease control rate of 70%; this currently fails to meet the ESMO Magnitude of Clinical Benefit Scale criteria and as such cannot be placed in tier 1b. As there are no data in ACC outside of NOTCH mutant patients, we cannot comment on there being a higher response rate in this setting (2b). As such, this match was given a rank of 4a [50,60].

PIK3R1 was mutated in 13% (4/32) and has been shown to cause hyperactivation of the PI3K pathway [45], indicating a potential role for AKT1 inhibition. In total, 9% (3/32) had alterations in genes involved in the DNA damage repair pathway that are thought to sensitize one to PARP inhibitors [51].

Previously undetected alterations in MET, ERBB2 and PTEN were detected, even though these genes were included in the primary analysis with the 24-gene panel. The alteration in MET was an amplification, which would not be detected by the 24-gene panel. The ERBB2 mutation was detected in the 24-gene panel but was not identified as being significant; this reflects the fact that there may be disagreement in approaches to variant calling. Finally, the PTEN alteration used in the secondary analysis used a tumour sample from a metastatic site, whereas the initial analysis in which this was not detected used a different sample from a primary site. This observation reflects the potential for discordance between mutations identified from primary and metastatic sites.

## 4. Discussion

This study sought to evaluate the clinical utility of tumour profiling by DNA-based NGS when applied to a cohort of patients with recurrent or metastatic salivary gland cancer being evaluated for clinical trials. We found, using a focused (24-gene) NGS panel, that genomic alterations were identified in 27% of patients with potential biomarker-matched clinical trial therapies available for 14% (25/175). The utility of this approach in identifying matched trial therapies was lower for ACC (7%) compared with other histological subtypes (30%), with the greatest utility seen for salivary duct carcinoma (69%). For ACC, broader (>325-gene) NGS panels provided additional utility in identifying matched clinical trial therapies, identifying matched trial therapies in 40% of patients in whom no alterations were identified using the focused panel. For this, we used a predesigned commercially available platform that is clinically and analytically validated for all solid tumours and has FDA approval as a companion diagnostic.

The 24-gene panel utilized was developed for pan-tumour use, in line with the United Kingdom National Genomic Test Directory for somatic mutations in adult solid tumours. This study found that 62% (15/24) of genes in our focused NGS panel are currently being investigated for their potential value as predictive biomarkers in ongoing trials in the UK. A total of 50% (12/24) of these genes are recognised by the FDA as predictive biomarkers or standard-of-care, and as positive predictive biomarkers of response in other tumour types other than salivary gland cancer. In this study, we used the ESMO Scale for Clinical Actionability of Molecular Targets (ESCAT) to determine the level of evidence supporting the match between genomic alteration and drug trial therapy. ESCAT breaks down evidence based on trial types, with prospective trials being evidence level 1, and this is further subdivided based on whether it was randomised (1a), nonrandomised (1b) or a basket trial (1c). Furthermore, ESCAT takes into account whether there was a benefit to overall survival (1a) or overall response rate (2b) [22]. Similar scoring systems such as OncoKB can be more ambiguous on these counts, placing a heavier focus on drug and biomarker approval for use, with nonapproved drugs being classified as “showing clinical benefit”. OncoKB provides a web-based resource which allocates biomarker evidence levels automatically; however, this is of more utility in common cancers, and only provides an outline as not all genes are currently included [21].

Mutations in TP53 and PIK3CA were the most frequently identified findings in this cohort. Pathogenic TP53 mutations were the most prevalent alteration identified in our focused panel, being present in 19% (34/175) of our cohort. We have previously shown TP53 mutation to be a negative prognostic factor in ACC [61]. There have, however, been no SGC specific studies investigating the predictive significance of TP53 mutations. Preclinical studies in cell lines other than SGC showed increased response rates to Wee1 inhibitors [62], but this has yet to translate into the clinic. An NCI-MPACT study, which was open to all solid tumour types, including SGC, investigated the Wee1 inhibitor Adavosertib in combination with carboplatin in a TP53-mutant cohort, but reported no significant responses [63]. Studies on ACC models have indicated that ATR inhibitors may have some benefit in ACC as ATR is downstream of MYB [48], paired with data from preclinical studies in other tumour types that have demonstrated that treatment with ATR inhibitors in TP53-mutant tumours results in increased radiotherapy and chemotherapy sensitisation [42], providing a rationale for the enrolling patients whose tumours harbour TP53 mutations in trials such as NCT03669601. However, there are more recent counterbalancing data that indicate that radiosensitisation is independent of TP53 [64].

PIK3CA was the second most prevalent finding, with 11/175 patients having mutations conferring increased activity. While there have been no clinical trials investigating PI3K specifically in SGC, six ACC patients were treated with PI3K inhibitors within basket trials: 5/6 patients had stable disease at 2 months, while one patient had a partial response by RECIST 1.1 criteria. It is, however, difficult to draw many conclusions due to the small sample size and very limited follow up reported [38]. There have been no SGC-specific preclinical studies demonstrating the predictive significance of PIK3CA mutation status. PIK3CA is an established predictive biomarker for treatment with Alpelisib plus fulvestrant in hormone receptor-positive breast cancer. Alpelisib as a monotherapy in PIK3CA mutated breast cancer did not show significant clinical benefit [65]. Trials of AKT1 inhibitors are also recruiting patients with activated PIK3CA (NCT NCT01226316, NCT02338622); these are also recruiting patients with PTEN loss, and with AKT1 mutations.

Although biomarker-led drug therapies are providing treatment options for patients with salivary gland cancers, this further subdivision of histopathological entities on the basis of molecular profiling exacerbates the challenges related to the rare nature of salivary cancers. Further subdividing an already small population for trial recruitment may pose a significant problem in obtaining adequate patient numbers to determine true benefits.

Some of the most clinically meaningful changes associated with biomarker-directed therapy development have been reported in HER2-overexpressing/ERBB2-amplified salivary duct carcinoma with HER2-targeted therapies [6,10,11]. Now, there is clear evidence of efficacy in the recurrent or metastatic setting, and ongoing trials are now evaluating HER2-targeted therapy as an adjuvant to surgery in the curative setting (NCT04620187 [6]). In addition, in recurrent or metastatic androgen receptor-overexpressing salivary duct carcinoma, now there is clear evidence of clinical benefit from androgen deprivation therapy [7] or combined androgen blockade [8]; there is also an emerging rationale for a clinical trial of ADT/CAB in the adjuvant setting for AR-overexpressing salivary duct carcinoma at high risk of disease recurrence.

Within ACC, NOTCH-activating mutations are a promising target; however, gamma secretase inhibitors such as AL101 have yet to show significant clinical benefit, being characterised by the EMSO magnitude of clinical benefit scale. However, the medium- and long-term outcomes of the phase 2 study (NCT03691207) are yet to be published. AL101 had an OR of 14% and a disease control rate (DCR) of 68% [50,60]. In NOTCH-activated ACC, which carries a significantly worse prognosis than NOTCH wildtype ACC [66,67] this DCR may translate into a meaningful clinical benefit.

In addition to genomic alterations that have current clinical utility, we have also described alterations which are currently classed as level X, meaning there is a lack of evidence of actionability. TERT promoter mutations were classified as X, and were found in 22% of ACC patients sequenced with the extended panel. While it provides no current benefit for treatment selection, previous studies have shown TERT to be a positive prognostic factor [23]. BCOR was similarly classified as X; however, BCOR is frequently mutated in haematological malignancies [68] and, as such, is likely to be investigated further and may become targetable in the future. As the median survival for recurrent or metastatic ACC is in excess of 5 years, it is not inconceivable that BCOR-targeting therapies could be in the clinic in that timeframe.

In this study, tumour tissue was analysed from either metastatic deposits or sites of local recurrence collected as part of their routine diagnostic or treatment. However, analysis from a single biopsy from a primary tumour or of a single metastatic site does not reliably cover the expected intra-patient tumour heterogeneity. For future studies, one approach to increase the likelihood of capturing intra-patient heterogeneity would be to include multiple biopsies or multiple time-points from individual patients. The sequencing of circulating tumour DNA (ctDNA) has been shown to be viable in the clinical setting with good concordance in variant calling with matched tumour samples. For example, the UK TARGET study identified actionable mutations in 41% (41/100) of patients through this method in a mixed cancer cohort, 25% (11/41) of which were treated in matched clinical trials (Rothwell et al.). Future prospective studies are planned in SGC which utilise sequencing of (ctDNA) to provide a minimally invasive and “current” picture of variants [69].

Furthermore, tissue samples for analysis were requested to meet the minimum tumour content of 20% for analyses, for which our panel was validated to detect a variant allele frequency of 4%. However, the exact tumour content of samples was not routinely recorded for all cases which we recognize as a limitation of this study. Although the risk of omitting internal validation of tumour content is the under-calling of mutations through sequencing of an increased percentage of normal tissue DNA, our detected frequencies are however in line with publicly available datasets [19,20].

While we focused on NGS though targeted gene panels within our study, whole genome (WGS) and whole exome sequencing (WES) could also be utilized in this setting. There is potential additional utility using these approaches to identify broader mutation signatures. WGS and WES also provide greater coverage capturing mutations in areas that may become clinically significant as our understanding of the underlying tumour biology increases. However, the additional data generated by WGS/WES approaches due to this broader coverage and additional sequencing steps, through sequencing matched normal DNA, require additional bioinformatics analyses. This plus the additional analytical reagents required make WGS and WES more expensive than targeted NGS panels. This contrasts with targeted NGS panels covering specific clinically important coding and noncoding regions. As such, required read depths can be achieved with minimal reagents and can be interpreted with simpler bioinformatics pipelines; however, this is at the cost of a limitation of utility for broader discovery research. The impact of mutations in genes involved in epigenetic regulation such as BCOR, can be difficult to predict due to the myriad of pathways that are regulated through epigenetics. Microarrays can be used for detecting variations in gene expression levels. This could be explored in future analyses to assess the impact of detected somatic mutations on the transcriptome. While BCOR is not currently targetable, mutations in this gene may result in overexpression a targetable protein.

A further barrier to the clinical application of the genomic findings is the limited availability of clinical trials recruiting in the UK. Relatively few sites are actively recruiting to each individual trial providing a practical or geographical barrier to clinical trial recruitment. For example, there were no available clinical trials accepting salivary gland cancer patients for the approved anti-EGFR therapies, such as afatinib and erlotinib or osimertinib within the UK. The UK is lacking a large tumour-agnostic trial such as NCI-MATCH(NCT02465060) or CAPTUR (NCT03297606). However, this situation may improve as the TAPISTRY trial (NCT04589845) is set to open for recruitment in the UK, enabling access to a selection of matched trial therapies.

The success of NTRK-targeted therapies in patients with secretory carcinoma harbouring NTRK gene fusions represents a significant step forward for genetic based approaches in SGC. These patients were not represented in our cohort, possibly due to the lower frequency of recurrence in this subtype in comparison to other salivary gland cancer types [70].

## 5. Conclusions

Tumour profiling with targeted DNA panels provides valuable information to help provide a rationale for enrolling patients in early phase trials. This approach showed the most utility in salivary duct carcinoma. Expanded sequencing with >325-gene panels provided added utility in ACC and identified the deregulation of transcriptional regulation with EP300, KDM6A and ARID1A mutations, in addition to the classically reported MYB translocations.

## Figures and Tables

**Table 1:** Clinical characteristics of patients with salivary gland cancer undergoing genomic profiling. Abbreviations: NGS. Next-generation sequencing; ACC (Adenoid Cystic Carcinoma); Acinic (Acinic Cell Carcinoma); Adeno (Adenocarcinoma); ExPleo (Carcinoma ex Pleomorphic Adenoma); SDC (Salivary duct carcinoma); MyoE (Myo-epithelial Carcinoma); MEC (Mucoepidermoid carcinoma); NEC (Neuro-endocrine carcinoma); Secretory (Secretory carcinoma); NUT (NUT carcinoma).

Characteristic	Subgroup	Total		NGS Successful		NGS Failed	
		Number	Percentage	Number	Percentage	Number	Percentage
**All Patients**		209	100	175	84	34	16
**Sex**	**Female**	113	54	93	53	20	59
	**Male**	96	46	82	47	14	41
**Age**	**Median**	51		51		51	
	**Range**	13–78		13–77		23–78	
**Site**	**Minor**	93	44	75	43	18	53
	**Major**	116	56	100	57	16	47
**R/M disease**	**Yes**	190	91	159	91	31	91
	**No**	19	9	16	9	3	9
**Metastatic sites**	**Local**	78	37	56	32	22	65
	**Lung**	121	58	99	57	22	65
	**Bone**	37	18	32	18	5	15
	**Liver**	22	11	22	13	0	0
	**Other**	24	11	21	12	3	9
**Subtypes**	**ACC**	142	68	115	66	27	79
	**Adeno**	18	9	15	9	3	9
	**SDC**	17	8	16	9	1	3
	**Acinic**	9	4	8	5	1	3
	**ExPleo**	8	4	8	5	0	0
	**MEC**	7	3	6	3	1	3
	**MyoE**	5	2	4	2	1	3
	**NEC**	1	1	1	1	0	0
	**Secretory**	1	1	1	1	0	0
	**NUT**	1	1	1	1	0	0

**Table 2:** Components of focused NGS panel and matched drug therapies. Biomarkers were classified based on ESCAT. The ESCAT score is attributed to specific gene variants as outlined in the original papers referenced. Only evidence for point mutations and small insertions and deletions are included. Other genetic alterations such as copy number variations are not included as they were not analysed in our panel.

GENE	ESCAT	Open Biomarker Selected Trials	Drug (Targeted Pathway)
AKT1	3a [24]	NCT03673787	Ipatasertib (AKT1 inhibitor)
		NCT01226316, NCT02338622	AZD5363 (AKT1 inhibitor)
AR	4a [25]	No trial	-
ALK	3a [26]	No trial	-
BRAF	3a [27]	NCT02407509	RO5126766 (Raf/MEK inhibitor)
CTNNB1	4a [28]	No trial	-
DDR2	4a [29]	No trial	-
EGFR	3a [30]	NCT04259450	AFM24 (anti-EGFR antibody)
ERBB2	3a/3b [6,10]	NCT01953926	Neratinib (Pan-Her inhibitor)
		NCT03410927	TAS0728 (Her-2 inhibitor)
FGFR2	4a [31]	NCT02052778, NCT04189445	Futibatinib (FGFR inhibitor)
		NCT04083976	Erdafitinib (FGFR inhibitor)
		NCT03822117	Pemigatinib (FGFR inhibitor)
GNA11	4a [32]	No trial	-
GNAQ	4a [32]	No trial	-
IDH1	3a [33]	NCT03684811	Olutasidenib (IDH1 inhibitor)
IDH2	3a [34]	No trial	-
KIT	3a [35]	NCT02571036	Ripretinib (KIT/PGDRa inhibitor)
KRAS	4a [27,36]	NCT02407509	RO5126766 (Raf/MEK inhibitor)
MAP2K1	3a [36]	NCT02407509	RO5126766 (Raf/MEK inhibitor)
MET	3a [37]	NCT02925104	Capmatinib (MET inhibitor)
NRAS	3a [36]	NCT02407509	RO5126766 (Raf/MEK inhibitor)
PDGFRa	3a [35]	NCT02508532	Avapritinib (PDGFRa inhibitor)
PIK3CA	2b [38]	NCT01226316, NCT02338622	AZD5363 (AKT1 inhibitor)
		NCT03006172	GDC-0077 (Pi3K inhibitor)
PTEN	3a [39]	NCT03673787	Ipatasertib (AKT1 inhibitor)
		NCT01226316, NCT02338622	AZD5363 (AKT1 inhibitor)
RET	3a [40]	NCT03037385	Pralsetinib (RET inhibitor)
		NCT03157128	Selpercatinib (RET inhibitor)
STK11	4a [41]	No trial	-
TP53	4a [42]	No trial	-

**Table 3:** Full list of significant alterations as classified by CanVIG [16,17]. Grouped by gene. ACC—adenoid cystic carcinoma; Acinic—acinic cell carcinoma, ADENO—adenocarcinoma not otherwise specified; ExPleo Carcinoma—expleomorphic adenoma; MEC—mucoepidermoid carcinoma; SDC—salivary ductal carcinoma.

Patient	Subtype	Variant	Patient	Subtype	Variant
ACC1	ACC	TP53 c.832C > T p.(Pro278Ser) 17% reads	ACC16	ACC	PIK3CA c.1633G > A p.(Glu545Lys) 11% reads
ACC3	ACC	TP53 c.814G > T p.(Val272Leu) 4% reads	ACC19	ACC	PIK3CA c.1258T > C p.(Cys420Arg) 18% reads;
ACC10	ACC	TP53 c.328delC p.(Arg110ValfsTer13) 6% reads	ACC19	ACC	PIK3CA c.1633G > A p.(Glu545Lys) 8% reads
ACC13	ACC	TP53 c.1146delA p.(Lys382AsnfsTer40) 29% reads	ACINIC25	ACINIC	PIK3CA c.3140A > G p.(His1047Arg) 23% reads
ACC14	ACC	TP53 c.375G > A p.(Thr125Thr) 28% reads	ADENO32	ADENO	PIK3CA c.1636C > A p.(Gln546Lys) 36.41% reads
ACC15	ACC	TP53 c.584T > C p.(Ile195Thr) 7% reads	ExPleo35	ExPleo	PIK3CA c.1633G > A p.(Glu545Lys) 39% reads
ACC18	ACC	TP53 c.373A > G p.(Thr125Ala) 14% reads	SDC44	SDC	PIK3CA c.1624G > A p.(Glu542Lys) 13% reads
ACC19	ACC	PIK3CA c.1633G > A p.(Glu545Lys) 8% reads	SDC45	SDC	PIK3CA c.1093G > A p.(Glu365Lys) 20% reads
ACC21	ACC	TP53 c.329G > C p.(Arg110Pro) 50% reads	SDC46	SDC	PIK3CA c.3140A > G p.(His1047Arg) 13% reads
ACC22	ACC	TP53 c.824G > A p.(Cys275Tyr) 4% reads	SDC47	SDC	PIK3CA c.1624G > A p.(Glu542Lys) 13% reads
ACC22	ACC	TP53 c.467G > C p.(Arg156Pro) 8% read	SDC49	SDC	PIK3CA c.3140A > G p.(His1047Arg) 10% reads
ACC24	ACC	TP53 c.1010G > A p.(Arg337His) 13% reads	SDC52	SDC	PIK3CA c.3140A > G p.(His1047Arg) 51% reads
ACINIC25	ACINIC	TP53 c.742C > T p.(Arg248Trp) 4% reads	ACC1	ACC	AKT1 c.49G > A p.(Glu17Lys) 4% reads
ACINIC26	ACINIC	TP53 c.818G > T p.(Arg273Leu) 35% reads	SDC54	SDC	AKT1 c.49G > A p.(Glu17Lys) 22% reads
ADENO27	ADENO	TP53 c.794T > G p.(Leu265Arg) 37% reads	SDC56	SDC	AKT1 c.49G > A p.(Glu17Lys) 19% reads
ADENO28	ADENO	TP53 c.637C > T p.(Arg213Ter) 26% reads	ACC5	ACC	PTEN c.829dup p.(Thr277AsnfsTer21) 9% reads
ADENO29	ADENO	TP53 c.626_627delGA p.(Arg209LysfsTer6) 30% reads	ACC7	ACC	PTEN c.750_765dup p.(Glu256TrpfsTer2) 52% reads
ADENO30	ADENO	TP53 c.481G > A p.(Ala161Thr) 22% reads;	ACC12	ACC	PTEN c.16A > T p.(Lys6Ter) 62% reads
ExPleo34	ExPleo	TP53 c.686_687dupGT p.(Thr230ValfsTer18) 38% reads	ACC17	ACC	PTEN c.686C > G p.(Ser229Ter) 12% reads
ExPleo35	ExPleo	TP53 c.991C > T p.(Gln331Ter) 44% reads;	ADENO31	ADENO	PTEN c.382A > C p.(Lys128Gln) 7% reads
ExPleo36	ExPleo	TP53 c.750_754dupCATCC p.(Leu252ArgfsTer95) 20% reads	MEC40	MEC	PTEN c.634 + 5G > T 42% reads
ExPleo37	ExPleo	TP53 c.989T > G p.(Leu330Arg) 26% reads	ACC4	ACC	EGFR c.2369C > T p.(Thr790Met) 31% reads
MEC38	MEC	TP53 c.828delC p.(Cys277ValfsTer68) 7% reads	ExPleo36	ExPleo	ERBB2 c.2305G > C p.(Asp769His) 81% reads
MEC39	MEC	TP53 c.733G > A p.(Gly245Ser) 15% reads	SDC43	SDC	ERBB2 c.929C > T p.(Ser310Phe) 34% reads
MEC41	MEC	TP53 c.460_462delGGC p.(Gly154del) 60% reads	SDC50	SDC	ERBB2 c.2305G > C p.(Asp769His) 26% reads
SDC42	SDC	TP53 c.839G > C p.(Arg280Thr) 14% reads	ACC11	ACC	BRAF c.1397G > A p.(Gly466Glu) 27% reads
SDC43	SDC	TP53 c.993 + 1G > T 41% reads	SDC45	SDC	BRAF c.1780G > A p.(Asp594Asn) 20% reads;
SDC45	SDC	TP53 c.374C > G p.(Thr125Arg) 22% reads	ADENO27	ADENO	KRAS c.436G > A p.(Ala146Thr) 8% reads
SDC48	SDC	TP53 c.841G > C p.(Asp281His) 39% reads.	ACC6	ACC	CTNNB1 c.134C > T p.(Ser45Phe) 23% reads
SDC50	SDC	TP53 c.377_380delACTC p.(Tyr126SerfsTer43) 18% reads			
SDC52	SDC	TP53 c.626_627delGA p.(Arg209LysfsTer6) 43% reads			
SDC53	SDC	TP53 c.919 + 1G > C 25% reads			
SDC54	SDC	TP53 c.650T > G p.(Val217Gly) 15% reads			
SDC55	SDC	TP53 c.452C > G p.(Pro151Arg) 21% reads			
SDC56	SDC	TP53 c.912delT p.(Lys305SerfsTer40) 17% reads			

## Data Availability

The data presented in this study are available on request from the corresponding author. The data are not publicly available due to the requirement to uphold the data sharing with relevant approved researchers as stipulated in the ethical approval.

## Supplementary Materials

The following supporting information can be downloaded at: https://www.mdpi.com/article/10.3390/cancers14051133/s1, Table S1: panel coverage, Table S2: ESCAT ref, Table S3: Variants.
